# AGE GROUP DIFFERENCES IN VIEWS ON ONE’S OWN OLDER ADULTHOOD

**DOI:** 10.1093/geroni/igac059.1602

**Published:** 2022-12-20

**Authors:** Shelbie Turner, Shannon Jarrott, Karen Hooker

**Affiliations:** Weill Cornell Medical College, Pitman, New Jersey, United States; The Ohio State University, Columbus, Ohio, United States; Oregon State University, Corvallis, Oregon, United States

## Abstract

Most of the current scholarship on views on one’s aging focuses on middle and older adulthood, prompting lifespan theorists to call for studies about views on one’s aging throughout the entire lifespan (Kornadt et al., 2020). In this study, we established a factor structure to Kornadt et al.’s (2020) Domain Specific Future Selves Scale, which prompted respondents to rate their thoughts on their self in old age across 9 different items. We then utilized measurement invariance testing to analyze how the factor structure differed between 433 young (18-39), 485 middle-aged (40-64), and 286 older adults (65 and older). Via Exploratory and Confirmatory Factor Analyses, three latent factors emerged, constituting future older selves in the domain of social lives (a=0.78), work and finances (a=0.62), and physical health (a=0.76). The model maintained configural (RMSEA = 0.06, CFI = .96; TLI = .95), metric (RMSEA = 0.06, CFI = .97; TLI = .96), and scalar (RMSEA = 0.06; CFI = .96; TLI = .95) invariance across all three age groups. Latent mean testing revealed that older adults thought more positively about their future social lives (p=0.005), work and finances (p=0.05), and health (p=0.02) than did younger adults, and more positively about their social lives (p=0.001) and health (p=0.05) than middle-aged adults. Results from this study offer additional psychometric validation of a measure of future selves that researchers can use across the lifespan, and deeper theoretical understanding of how domain-specific views on one’s own older adulthood might differ between age groups.

